# *In-situ* fabrication of self-supported cobalt molybdenum sulphide on carbon paper for bifunctional water electrocatalysis

**DOI:** 10.1016/j.heliyon.2024.e31108

**Published:** 2024-05-10

**Authors:** Yuting Yao, Yuhan Liu, Juhun Shin, Shenglin Cai, Xinyue Zhang, Zhengxiao Guo, Christopher S. Blackman

**Affiliations:** a Department of Chemistry, University College London, 20 Gordon Street, London, WC1H 0AJ, UK; b Yusuf Hamied Department of Chemistry, University of Cambridge, Lensfield Road, Cambridge, CB2 1EW, UK; c Department of Chemistry, HKU-CAS Joint Laboratory on New Materials, University of Hong Kong, Hong Kong SAR, 999077, China; d HKU Zhejiang Institute of Research and Innovation, Hangzhou, 311305, China

**Keywords:** CoMoS phase, Electrochemical water splitting, Heterostructure coupling, Synergistic effect, Aerosol assisted chemical vapour deposition

## Abstract

The fabrication of highly efficient yet stable noble-metal-free bifunctional electrocatalysts that can simultaneously catalyse both hydrogen evolution reaction (HER) and oxygen evolution reaction (OER) remains challenging. Herein, we employ the heterostructure coupling strategy, showcasing an aerosol-assisted chemical vapour deposition (AACVD) aided synthetic approach for the *in-situ* growth of cobalt molybdenum sulphide nanocomposites on carbon paper (CoMoS@CP) as a bifunctional electrocatalyst. The AACVD allows the rational incorporation of Co in the Mo–S binary structure, which modulates the morphology of CoMoS@CP, resulting in enhanced HER activity (ŋ_10_ = 171 mV in acidic and ŋ_10_ = 177 mV in alkaline conditions). Furthermore, the CoS_2_ species in the CoMoS@CP ternary structure extends the OER capability, yielding an ŋ_100_ of 455 mV in 1 M KOH. Lastly, we found that the synergistic effect of the Co–Mo–S interface elevates the bifunctional performance beyond binary counterparts, achieving a low cell voltage (1.70 V at 10 mA cm^−2^) in overall water splitting test and outstanding catalytic stability (∼90 % performance retention after 50-/30-h continuous operation at 10 and 100 mA cm^−2^, respectively). This work has opened up a new methodology for the controllable synthesis of self-supported transition metal-based electrocatalysts for applications in overall water splitting.

## 1 Introduction

Accelerating global warming and its associated climate change has created a strong impetus for the development of clean and sustainable alternative energy vectors to replace the currently widely used fossil fuels, to attain net-zero CO_2_ emissions in the medium term. Molecular hydrogen (H_2_) has been regarded as such an alternative due to its high energy density and conversion efficiency, and potentially zero-carbon emission (dependent on production). Electrochemical water splitting provides the ability to convert renewable but intermittent energy, such as solar or wind, into hydrogen that can be stored for later use [[1], [2], [3]], however this process, consisting of cathodic hydrogen evolution reaction (HER) and anodic oxygen evolution reaction (OER), is kinetically rather sluggish, which necessitates relatively high overpotential or effective electrocatalysts to overcome the energy barriers [4,5].

Currently, platinum acts as the most efficient catalyst for HER, while iridium oxides remain one of the benchmark catalysts for OER [6,7]. Meanwhile, ruthenium, through a series of rational modifications, has emerged as one of the most promising catalysts for overall water splitting in wide PH range [[8], [9], [10], [11]]. Although effective, these electrocatalysts are expensive with limited reserves on Earth, which restricts their large-scale application [6]. To overcome this problem, intensive efforts have been devoted to developing Earth-abundant substitutes, for example first-row transition metal-based compounds [[12], [13], [14], [15], [16], [17]] or metal-free nanostructures [18]. However, the majority of previously reported non-noble metal electrocatalysts are only efficient for a single half-reaction, either HER or OER and/or only suitable in either acidic or alkaline media, and hence have limited efficiency in overall water electrolysis.

To achieve the bifunctionality of electrocatalysts, major attention has been paid to the development of heterostructures of transition-metal-based catalysts, which involves the rational incorporation of two or more electroactive species towards HER and/or OER [[19], [20], [21], [22], [23], [24], [25]]. These structures can not only achieve bifunctional features but also provide synergistic effects for improving the catalytic activity [[26], [27], [28]]. For instance, Zhu et al. [20] developed a Co_9_S_8_@MoS_2_ core-shell system on carbon nanofibers for electrocatalytic water splitting. However, the fabrication of heterogeneous composites, typically via hydro/solvothermal, electrodeposition, vapour deposition, vacuum filtration or their combinations [29], generally involves complicated multi-step procedures. Further, the majority of previous materials typically exist in the form of powder which needs to be cast onto the current collector using binders, which may hinder active sites, lower mass transport [29], and weaken the long-term stability [30].

To address these challenges, innovative approaches such as microwave-assisted synthesis [[31], [32], [33]] and aerosol-assisted chemical vapour deposition (AACVD) [34] are employed to synthesize materials in a simple and rapid manner. In contrast to conventional CVD, which demands the use of highly volatile precursors, AACVD is a solution-based technique that allows the growth of metals or metal oxides at high deposition rates whilst relaxing the requirement of precursor volatility at relatively low (evaporation) temperature. This feature allows a wide choice of precursors, which greatly facilitates the synthesis of versatile self-supported single/multi-component electrocatalysts with controllable morphologies and compositions in a scalable manner [35,36]. Recently, Ehsan et al. have reported the deposition of several metal-based materials on nickel foam (NF) via AACVD and their use as electrocatalysts [[37], [38], [39], [40]], including Pt films and nanoporous Pd for HER, and Pd–CoO films and Au- supported CoO_x_ films for OER. However, those works mainly focused on noble metal electrocatalysts with single functionality, either HER or OER.

Herein, we report an AACVD-based approach for *in-situ* growth of CoMoS nanocomposites on carbon paper (CoMoS@CP), creating highly stable and efficient bifunctional electrocatalysts for overall water splitting. By adopting the heterostructure coupling strategy, CoMoS@CP synthesized by rational incorporation of HER-active MoS_2_ and OER-active CoS_2_ species requires 171 and 177 mV to catalyse HER under acidic (0.5 M H_2_SO_4_) and alkaline (1 M KOH) conditions at 10 mA cm^−2^, respectively; it also exhibits efficient OER activity in an alkaline medium, requiring only 455 mV overpotential to attain a high current density of 100 mA cm^−2^. Furthermore, we demonstrated the ability to use the AACVD-deposited CoMoS@CP as a bifunctional electrocatalyst for overall water splitting, yielding a cell voltage of 1.70 V at 10 mA cm^−2^. The long-term stability tests show that the CoMoS@CP can sustain catalytic activity for 50 h at 10 mA cm^−2^ with only a 10.3 % performance loss and for 30 h at 100 mA cm^−2^ with an 11.5 % loss.

## 2 Experimental section

### 2.1 Fabrication of CoMoS@CP electrocatalyst

#### 2.1.1 Aerosol assisted chemical vapour deposition of CoMoO on CP

In a typical reaction, carbon paper was cut to 0.5 cm × 0.7 cm. The bottom 0.5 cm × 0.5 cm is the material deposition area, and the top 0.5 cm × 0.2 cm is left blank to be clamped by the electrode holder when carrying out the electrochemical measurement. The as-cut carbon paper was then washed with methanol and acetone and dried under airflow. After placing the clean carbon paper on the CVD reactor chamber, the chamber was heated to 275 °C under 500 sccm nitrogen flow. Meanwhile, 0.015 mmol molybdenum hexacarbonyl (Mo precursor) and 0.015 mmol cobalt(II) acetylacetonate (Co precursor) were dissolved in 15 mL acetone in separate flasks and sonicated in the water bath. Afterwards, the acetone solutions containing Mo precursor and Co precursor were atomised successively by the humidifier to deposit CoMoO on the carbon paper. After successful deposition, the reactor chamber was cooled down to below 100 °C under continuous nitrogen flow to safely obtain the as-synthesized carbon paper. The total number of moles of the precursors used was kept consistent at 0.03 mmol for all AACVD reactions, unless specified otherwise. Control samples CoO_x_@CP and MoO_x_@CP were prepared in the same manner with 0.03 mmol of either Co or Mo precursor in acetone during the CVD process.

#### 2.1.2 Sulphurisation of CoMoO on CP

The as-prepared intermediate CoMoO@CP was then sulphurised in the tube furnace at various annealing temperatures under nitrogen flow (200 sccm) for 5 h at the heating rate of 5 °C min^−1^. The carbon paper covered by CoMoO was placed in the middle of the tube furnace, while the combustion boat containing 1 g sulphur was placed 12 cm upstream of the nitrogen inlet. The CoMoO@CP was sulphurised at 450 °C, followed by a cooling step to room temperature to produce CoMoS@CP. Control samples, MoO_x_@CP and CoO_x_@CP, were sulphurised using the same process to generate MoS_x_@CP and CoS_x_@CP. The mass loading of the samples was estimated by weighing the CP before and after the two-step fabrication process.

#### 2.1.3 Materials characterisation

The morphological characterisation of the as-synthesized samples was carried out using field emission scanning electron microscope (Jeol JSM 7600 FEG-SEM) and transmission electron microscopy (Jeol 2100 TEM). The 10.13039/501100001838TEM samples were prepared by sonicating the CoMoS@CP in methanol in a water bath for 30 min, and then the delaminated CoMoS@CP from the bulk sample was dropped on the holey carbon-supported copper grids and air-dried. Energy-dispersive X-ray spectroscopy (EDS) was obtained by an Oxford Instruments X-MaxN 80-T Silicon Drift Detector (SDD) fitted to the SEM/TEM machine. The Grazing incidence x-ray diffraction (GIXRD) was used to acquire the phase identity of the nanocomposite-coated carbon paper. The x-ray source used for GIXRD is Cu Kα radiation (wavelength of 1.54 Å), and the scan range is 10°-65° with 0.05° per step every 2 s. The X-ray photoelectron spectroscopy (XPS) measurements were taken by Al Kα (1486.6 eV), Thermo Scientific, and the data was analysed using CasaXPS. The Raman spectra acquisition was carried out using Renishaw multiline spectrometer with signals excited at 514.5 nm.

### 2.2 Electrochemical measurements

All the electrochemical measurements were carried out using Gamry Instrument. A typical three-electrode electrochemical cell with two compartments separated by Nafion™ membrane was used throughout the electrochemical HER and OER tests. The as-synthesized nanocomposite-coated carbon paper was clamped as the working electrode without further treatments along with Ag/AgCl in 3 M KCl reference electrode in the same cell compartment. The graphite rod used as the counter electrode was placed in the other compartment. The benchmark 20 % Pt/C and RuO_2_ electrode sample were prepared by drop-casting the Nafion™ containing 20 % Pt/C or RuO_2_ slurry on carbon paper and air-dried (mass loading = 0.2 mg cm^−2^).

The as-prepared samples were activated by 20 cycles of cyclic voltammetry (CV) with scan rate of 50 mV s^−1^. The applied potentials for HER tests ranged from 0 V to −0.7 V in 0.5 M H_2_SO_4_, -0.4 V to −1.5 V in 1 M KOH, and 0 V–0.8 V OER tests in 1 M KOH, all referenced to Ag/AgCl. The potential *E* (vs. Ag/AgCl) was corrected with respect to reversible hydrogen electrode (RHE) using equation *E* (vs. RHE) = *E* (vs. Ag/AgCl) + 0.059 x pH + 0.1988. The electrocatalytic performance was then investigated using linear sweep voltammetry (LSV) at scan rate of 5 mV s^−1^, with 80 % iR compensation. The Tafel plot was constructed with log |*j*| vs. *η*, where *j* is the current density in mA cm^−2^ and *η* is the overpotential. The Tafel slope was derived from the gradient of the linear polarisation region of the plot. The catalytic stability of the samples was assessed by applying (±) 10 mA cm^−2^ over the samples for 24 h.

The overall water splitting capability was evaluated by constructing a two-electrode electrolyser with two identical 0.5 × 0.5 cm^2^ samples set as working and counter electrodes respectively. The current response was recorded with cell potential ranging from 0.2 to 2.2 V vs. Ag/AgCl with scan rate of 5 mV s^−1^. The OER happens at the working electrode and HER occurs at the counter electrode. The catalytic stability of the cell is assessed by applying 10 and 100 mA cm^−2^ over the electrodes for 50 and 30 h respectively.

## 3 Results and discussion

### 3.1 Fabrication and characterisation of CoMoS@CP electrocatalyst

**Fig. 1:**
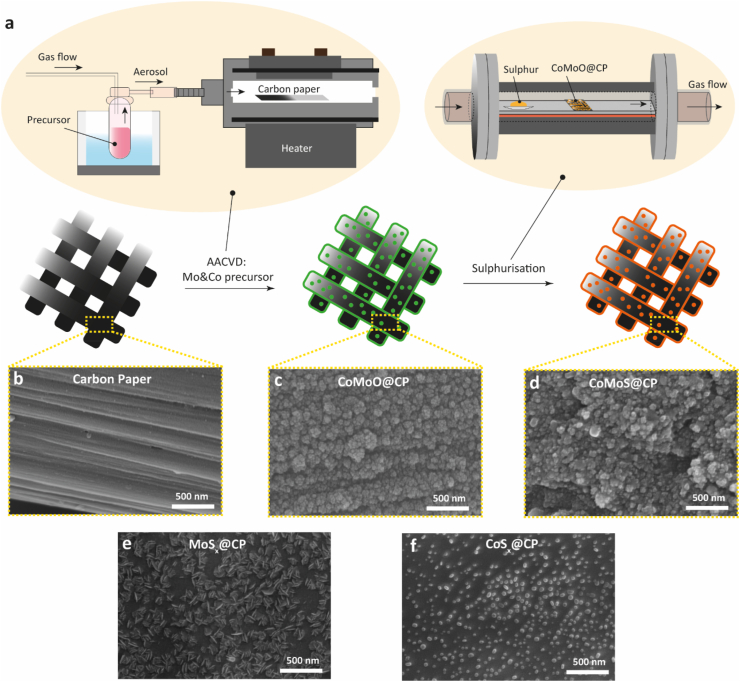
(a) Schematic representation of the two-step synthesis of CoMoS@CP. SEM images of (b) carbon paper, (c) CoMoO@CP, (d) CoMoS@CP, (e) MoS_x_@CP and (f) CoS_x_@CP.

The self-supported CoMoS@CP catalyst was fabricated *in-situ* on a commercial carbon paper (CP) via a facile two-step process (schematic representation shown in Fig. 1a). In the initial step, a typical AACVD reactor setup was utilised, comprising two main components: an aerosol generator (in this instance an ultrasonic humidifier) and a thermocouple-controlled reaction chamber. During the AACVD process, the precursor solution was nebulised to form aerosol droplets, which were subsequently transported to the reaction chamber with the assistance of a carrier gas (N_2_). At the optimal temperature, the vapourised precursor underwent heterogeneous decomposition on the surface of the substrate to form an adhesive coating [35]. In this work, after successive depositions employing the individual Mo and Co precursors, a layer of cobalt molybdenum oxide was deposited onto the carbon paper, denoted as CoMoO@CP. In the second step, this CoMoO@CP was sulphurised in a nitrogen atmosphere to obtain the cobalt molybdenum sulphide nanocomposites, termed as CoMoS@CP. For comparative purposes, separate Co or Mo sulphides were also deposited on carbon paper using the same process, designated as CoS_x_@CP and MoS_x_@CP, respectively.

The morphologies of obtained samples were characterised using field-emission gun scanning electron microscopy (FEG-SEM). As displayed in Fig. 1b and Fig. S1, the morphology for blank carbon paper exhibits typical weaving carbon fibres with porous structure in between. After the AACVD procedure, the CoMoO@CP composite appeared as uniformly dispersed nanoparticles with sizes between 20 and 50 nm (Fig. 1c). The uniformity was also verified by EDS mapping of Co and Mo (Fig. S2) where the atomic ratio was roughly 1:2. After sulphurisation, CoMoS composite retained similar morphology (shown in Fig. 1d and Fig. S3). Compared to the CoMoS@CP sample, MoS_x_@CP (SEM images in Fig. 1e and Fig. S4a) showed MoS_x_ nanoplates aligning vertically over the carbon paper and the observed atomic ratio of Mo and S was around 1:2, suggesting that the MoS_x_ formed is MoS_2_ [41]. The morphological differences between CoMoS@CP and MoS_x_@CP suggested that the uniformly incorporated Co species in CoMoO@CP, during the AACVD, inhibited the stacking of MoS_2_ nanoplates in the subsequent sulphurisation step, potentially enriching the exposure of the edge-terminated active sites [42]. In addition, the SEM images in Fig. 1f and Fig. S4b indicated that the CoS_x_ nanoparticles grown on the CP ranged from 10 to 50 nm. The atomic ratio of Co to S was roughly 1:2, implying the as-formed nanoparticles were CoS_2_.

**Fig. 2:**
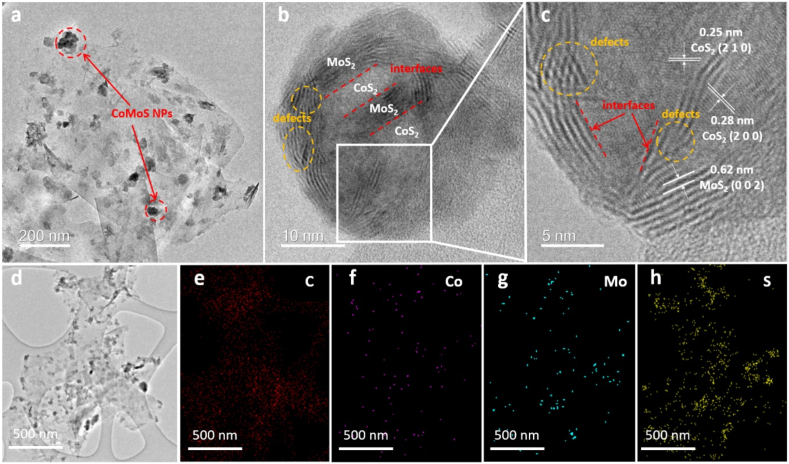
(a–d) TEM images of CoMoS@CP at different magnifications. The EDS mappings of (e) C, (f) Co, (g) Mo and (h) S for CoMoS@CP.

The morphologies and elemental compositions of the CoMoS@CP were further investigated using TEM and EDS mapping. As shown in Fig. 2a, the peeled CoMoS@CP exhibited evenly dispersed CoMoS nanoparticles with sizes consistent with those seen in the SEM images. Higher magnification TEM images (Fig. 2b and c) revealed the lattice spacings of 0.28 nm and 0.25 nm which can be attributed to (2 0 0) and (2 1 0) planes of CoS_2_ and a spacing of 0.62 nm which can be attributed to (0 0 2) planes of MoS_2_. Importantly, we also observed that the continuity of the MoS_2_ planes was truncated by the CoS_2_ planes, leading to the formation of ultrathin MoS_2_ sheets with an average of 4–6 layers. This interlacing of MoS_2_ and CoS_2_ planes resulted in abundant Co–Mo–S interfaces and lattice defects in CoMoS nanocomposites, which are assumed to facilitate the electrochemical activity [43]. Furthermore, the TEM image for the CoMoS@CP nanocomposites (Fig. 2d) and its corresponding EDS mapping (Fig. 2e–h) indicated that all the Co, Mo, and S were distributed homogeneously on CP and the atomic ratio is roughly 0.5:1.3:2.5.

**Fig. 3:**
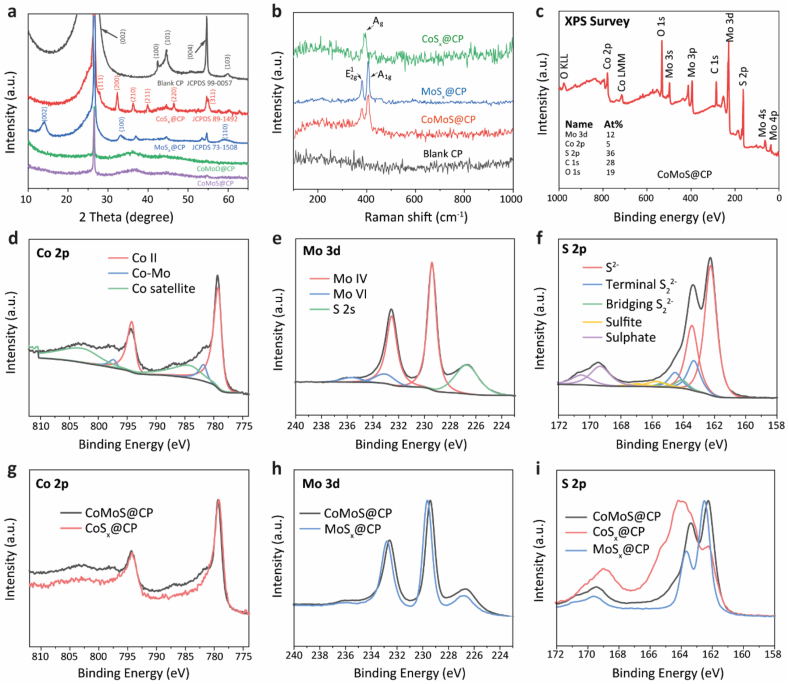
(a) XRD patterns of CoMoS@CP and control samples. The overall loading of the precursors was 1 mmol during the AACVD process for all samples to ensure the diffraction peaks are sufficiently significant to be identified. (b) Raman spectra for CoMoS@CP, CoS_x_@CP, MoS_x_@CP and blank carbon paper. (c) XPS survey of CoMoS@CP showing the atomic ratio of each element. Deconvoluted high-resolution spectra of (d) Co 2*p*, (e) Mo 3*d*, (f) S 2*p* for CoMoS@CP. The Co 2*p* spectrum was deconvoluted with background offsetting at higher binding energy to avoid overlapping between the Shirley background and the valley at ∼790 eV [48]. Min-max-normalised (g) overlay Co 2*p* spectra of CoMoS@CP and CoS_x_@CP, (h) overlay Mo 3*d* spectra of CoMoS@CP and MoS_x_@CP, (i) overlay S 2*p* spectra of CoMoS@CP, CoS_x_@CP and MoS_x_@CP.

X-ray diffraction (XRD) was used to examine the crystal phases of the as-obtained samples as shown in Fig. 3a. As a control, the XRD pattern of blank carbon paper showed peaks at 26.5°, 42.5°, 44.7°, 54.7° and 60.1°, corresponding to the (0 0 2), (1 0 0), (1 0 1), (0 0 4) and (1 0 3) diffraction peaks of graphite (JCPDS No. 99-0057) [44]. The XRD pattern for CoS_x_@CP showed diffraction peaks at 27.9°, 32.3°, 36.3°, 39.9°, 46.4° and 55.0°, which were assigned to (1 1 1), (2 0 0), (2 1 0), (2 1 1), (2 2 0) and (3 1 1) planes of CoS_2_ (JCPDS No. 41–1471, cubic) [45,46]. For the MoS_x_@CP sample, we observed diffraction peaks at 14.4°, 32.8° and 58.6°, implying the (0 0 2), (1 0 0) and (1 1 0) planes of MoS_2_, (JCPDS: 73–1508, hexagonal) [47]. However, compared to crystalline CoS_x_@CP and MoS_x_@CP, the XRD patterns of CoMoO@CP and CoMoS@CP both show broad features around 32°-37°, indicative of amorphous structures. The amorphous structure of CoMoO@CP is due to the low optimum deposition temperature of the AACVD process, which is insufficient to promote the long-range atomic ordering required for crystallinity. Subsequent sulphurisation of CoMoO@CP at 450 °C to form CoMoS@CP does not alter this amorphous character. This is attributed to the coupling between CoS_2_ and MoS_2_ during the sulphurisation process, which disrupts the formation of a crystalline structure. This coupling feature suggests the interfacial interaction of Co–Mo–S, which is also verified by the XPS results in the following discussion.

The coupling phenomenon was also verified by the Raman characterisation shown in Fig. 3b. There were no obvious Raman peaks observed for the blank CP in the range 100–1000 cm^−1^, while all Co and Mo containing samples displayed characteristic peaks between 380 and 410 cm^−1^. CoS_x_@CP showed a characteristic peak at Raman shift of 395 cm^−1^, which was attributed to the A_g_ vibration mode of CoS_2_ [49,50]. The broad feature observed at ∼300 cm^−1^ can be assigned to E_g_ and T_g_(1) vibration modes of CoS_2_ with relatively low intensities [49,50]. On the other hand, the MoS_x_@CP showed typical in-plane E^1^_2g_ and out-of-plane A_1g_ vibration modes of MoS_2_ indexed at Raman shifts of 380 and 407 cm^−1^, respectively. For the ternary sample CoMoS@CP, a combination of Raman signals of MoS_x_@CP and CoS_x_@CP were observed (broadening of MoS_2_ E^1^_2g_ and A_1g_ modes), indicating the successful coupling of the two species [51].

The surface of the as-obtained samples was examined using X-ray photoelectron spectroscopy (XPS). The XPS survey spectrum of CoMoS@CP showed the presence of Mo, Co, S, C and O with surface atomic ratios of Co 2*p*, Mo 3*d* and S 2*p* of 5 %, 12 % and 36 %, respectively (Fig. 3c), which roughly agrees with the EDS mapping results (1:2:6). The Co 2*p*, Mo 3*d* and S 2*p* high-resolution spectra of CoMoS@CP were deconvoluted and illustrated in Fig. 3d–f, as well as the overlays of CoS_x_@CP and MoS_x_@CP for comparison (Fig. 3g–i). As shown in Fig. 3d–g and Figs. S5a and a Co^2+^ 2*p* doublet was observed at 778.9 and 793.9 eV of both CoMoS@CP and CoS_x_@CP, originating from CoS_2_ [[52], [53], [54]]. However, compared to CoS_x_@CP, CoMoS@CP showed an additional doublet at binding energies of 781.5 and 797.1 eV in the Co *2p* region. This new feature suggests that the introduction of the more electronegative Mo leads to electron loss from Co, resulting in a higher oxidation state of Co and consequently, the appearance of new peaks at these higher binding energies, indicative of modified electronic states due to interfacial interaction between Co and Mo. The broad characteristic peaks at slightly higher energies than the Co(II) doublets were assigned as Co satellites. As for the Mo 3*d* region (Fig. 3e–h and Fig. S5c), the Mo^4+^ stemming from MoS_2_ was observed at 229.4 and 232.5 eV for CoMoS@CP [55]; energies were shifted ∼0.2 eV more negative as compared to that of the MoS_x_@CP (Fig. 3h). This observation suggested that the formation of Co–Mo–S interfaces involved charge transfer from the Co species to the Mo species [56,57]. Further, an extra doublet belonging to Mo^6+^ appeared at higher binding energies of 232.6 and 235.7 eV, which was ascribed to surface oxidation [43]. The S 2*s* peak overlapped with Mo 3*d* at 226.7 eV. The deconvolution of the S 2*p* spectrum of CoMoS@CP showed five chemical states at 162.3, 163.3, 164.1, 165.7 and 169.3 eV (Fig. 3f), which can be assigned to S 2*p*_3/2_ of sulphides (S^2−^), terminal disulphides (S_2_^2−^), bridging disulphides (S_2_^2−^), sulphites and sulphates, respectively [58]. In particular, the S_2_^2−^ originates from the unsaturated S atoms of the defective S edge structures and are catalytically active toward HER [23]. The formation of sulphates was due to surface oxidation. Notably, we observed all five of these S chemical environments for CoS_x_@CP with a smaller relative proportion of S^2−^ (Fig. 3i and Fig. S5b), while the S 2*p* spectrum of MoS_x_@CP was dominated by S^2−^ only (Fig. S5d). The increased proportion of unsaturated S_2_^2−^ ligands in CoMoS@CP compared to that of MoS_x_@CP is attributed to the incorporation of CoS_x_ species, i.e., the Co atoms in the Co–Mo–S interfaces generate S vacancies at/next to Co sites which are potentially more electroactive [59,60].

Taking together the morphological and structural analysis, the CoMoS@CP was characterised as defect-rich nanoparticles, consisting of both CoS_2_ and MoS_2_ phases with Co–Mo–S interfaces, uniformly deposited on carbon paper. The control samples CoS_x_@CP and MoS_x_@CP are denoted as CoS_2_@CP and MoS_2_@CP in the following sections after confirming their crystalline structures and elemental compositions using XRD, EDS and XPS.

### 3.2 HER and OER activity of CoMoS@CP

**Fig. 4:**
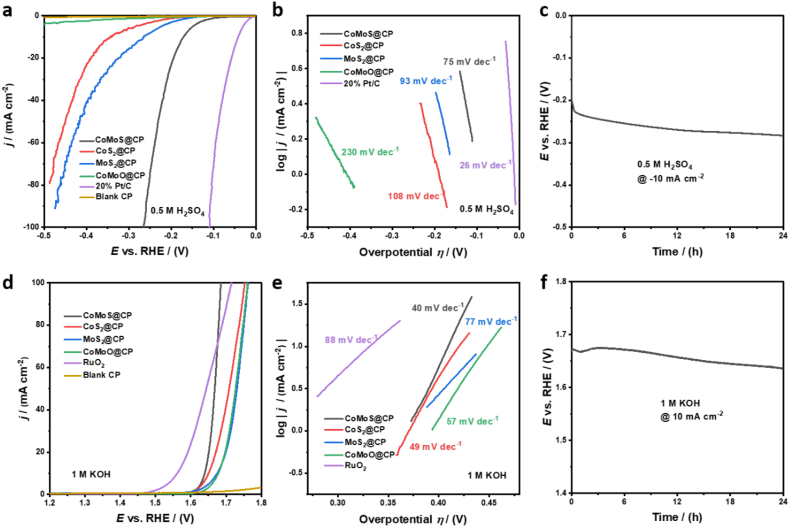
(a) Electrocatalytic HER polarisation curves of CoMoS@CP and control samples in 0.5 M H_2_SO_4_ and (b) the corresponding Tafel plots. (c) Chronopotentiometry scan at -‍10 mA cm^−2^ for 24 h to test the long-term catalytic stability of CoMoS@CP for HER in 0.5 M H_2_SO_4_. (d) Electrocatalytic OER polarisation curves of CoMoS@CP and control samples in 1 M KOH and (e) the corresponding Tafel plots. (f) Chronopotentiometry scan at 10 mA cm^−2^ for 24 h to test the long-term catalytic stability of CoMoS@CP for OER in 1 M KOH.

The HER performance of the CoMoS@CP electrocatalyst was measured in 0.5 M H_2_SO_4_ electrolyte by recording linear sweep voltammetry (LSV) polarisation curves in a typical three-electrode cell with iR-loss correction. For comparison, the HER activities of CoS_2_@CP, MoS_2_@CP, CoMoO@CP, bare CP and 20 % Pt/C were also tested. As shown in Fig. 4a (also in Table S1), the 20 % Pt/C shows excellent HER activity (with ŋ_10_ = 42 mV and ŋ_100_ = 110 mV) as expected. The CoMoS@CP exhibited the best HER activity among the as-synthesized electrocatalysts with overpotentials of 171 and 265 mV, respectively, to achieve 10 and 100 mA cm^−2^. In comparison, the mono-metal counterparts, CoS_2_@CP and MoS_2_@CP, showed much inferior HER activities with higher overpotentials of 343 and 265 mV, respectively, to reach 10 mA cm^−2^. Notably, the intermediate, CoMoO@CP and the blank CP substrate showed extremely poor HER activities, in which the current densities do not even reach 5 mA cm^−2^ at 500 mV overpotential. To explore the feasibility of CoMoS@CP as a potential bifunctional electrocatalyst for overall water splitting under alkaline conditions, we also tested its HER performance in 1 M KOH. As shown in Fig. S6, the HER performance of CoMoS@CP (ŋ_10_ = 177 mV) again outperformed its counterparts.

The Tafel slopes which reflect the inherent kinetics of electrochemical reactions were examined for these synthesized electrocatalysts. As shown in Fig. 4b, the Tafel slope for the CoMoS@CP nanocomposite was observed to be approximately 75 mV dec^−1^, which is considerably lower than those for CoS_2_@CP (108 mV dec^−1^), MoS_2_@CP (93 mV dec^−1^) and CoMoO@CP (230 mV dec^−1^). The slope for the CoMoS@CP electrocatalyst reveals that the rate-determining step is the Volmer step [61,62].

To gain further insight into the activity enhancement, electrochemical impedance spectroscopy (EIS) was performed. The Nyquist plots were recorded at −0.173 V vs. RHE and fitted using a Simplified Randles equivalent circuit as illustrated by the inset of Fig. S7, where R_s_ is the resistance of the electrolyte, CPE and R_ct_ represent constant phase element and charge transfer resistance of the electrocatalyst, respectively. Under the Simplified Randles model, the R_ct_ is determined by calculating the difference between high/low-frequency intercepts at the real axis [63]. As shown in Fig. S7, the estimated R_ct_ of CoMoS@CP (21.2 Ω) was significantly lower than that of CoS_2_@CP (253 Ω), MoS_2_@CP (139 Ω), and CoMoO@CP (4640 Ω), revealing the excellent charge-transfer capability of CoMoS@CP. The electrochemical active surface areas (ECSA) were also investigated as shown in Fig. S8. The double-layer capacitance (C_dl_) obtained for CoMoS@CP (4.4 mF cm^−2^) was significantly larger than that of the other prepared materials (Fig. S8f and Table S1). This indicated a larger electrochemical active surface area for CoMoS@CP, which accounted in part for its improved HER performance.

We also examined the long-term catalytic stability of the CoMoS@CP electrocatalyst in both electrolytes. As shown in Fig. 4c, the CoMoS@CP electrode in 0.5 M H_2_SO_4_ showed a potential drop of 60 mV to maintain 10 mA cm^−2^ during the 24-h stability test after an initial drop in potential during the first 30 min. While in 1 M KOH (Fig. S6c), after the 24-h test, CoMoS@CP retained around 90 % of its performance. Considering the low loading of CoMoS on CP (∼0.02 mg cm^−‍2^), the activity of CoMoS@CP is competitive with the similar Co–Mo–S ternary composites reported previously (Table S2), [20,47,51,[64], [65], [66], [67], [68]].

We attribute the enhanced HER performance of CoMoS@CP to the formation of Co–Mo–S interfaces. The *in-situ* AACVD process ensures the uniform incorporation of Co species to the Mo species, thus facilitating the formation of Co–Mo–S interfaces in the subsequent sulphurisation step. Typically, MoS_2_ adopts a truncated triangular morphology, with basal planes remaining HER inactive and edges dominated by HER active Mo (1 0 $\bar{1}$ 0) sites. When Co is introduced, it tends to substitute only the Mo atoms at the S ($\bar{1}$ 0 1 0) edge and bond with only four S atoms, resulting in partially unsaturated S atoms that serve as active sites for HER [59]. The resultant Co–Mo–S interface adopts a truncated hexagonal morphology, exposing HER active sites containing Mo (1 0 $\bar{1}$ 0) and Co–Mo–S ($\bar{1}$ 0 1 0) edges [42]. Furthermore, the perturbation of surface electronic distribution induced by Co–Mo–S enhances the free energy of proton adsorption, further improving HER activity [59,69].

It is important to examine the OER efficiency of electrocatalysts as it is another half-reaction of electrocatalytic water splitting. As compared to HER, OER typically displays sluggish reaction kinetics with a four-electron transfer process involved, which commonly requires a larger overpotential [13]. The capability of OER of all as-synthesized electrocatalysts were investigated in alkaline electrolyte (1 M KOH) and plotted in Fig. 4d. Among these noble-metal-free catalysts, the CoMoS@CP showed the best OER activity with the lowest overpotential of 409 mV at 10 mA cm^−2^, while values for CoS_2_@CP, MoS_2_@CP, CoMoO@CP were relatively higher at 420, 445 and 448 mV, respectively. The OER activity for the blank CP was almost negligible. For CoMoS@CP, only 80 mV extra overpotential was required to reach 10 mA cm^−2^ current density as compared to that of the standard reference, RuO_2_ (η_10_ = 330 mV). Notably, the overpotential required for CoMoS@CP at the higher current density of 100 mA cm^−2^ was 455 mV, which was 30 mV lower than that of RuO_2_ (η_100_ = 485 mV). These results indicate that the CoMoS@CP has faster conversion kinetics after reaching a certain overpotential. The fast reaction kinetics is also verified by the Tafel slopes in Fig. 4e. Especially, the Tafel slope of CoMoS@CP was 40 mV dec^−1^, suggesting that the anodic current increased exponentially with increasing overpotential. The Tafel values of CoS_2_@CP, MoS_2_@CP and CoMoO@CP were 49, 77 and 57 mV dec^−1^, respectively, all lower than that of RuO_2_ (88 mV dec^−1^). The Nyquist plots, shown in Fig. S9, revealed the lowest R_ct_ for CoMoS@CP electrode (5.35 Ω) compared to those of the other materials (7.75, 18.1, and 62.1 Ω for CoS_2_@CP, MoS_2_@CP and CoMoO@CP, respectively). The C_dl_ measured (shown in Fig. S10 and Table S3) for the CoMoS@CP was 6.7 mF cm^−2^, better than that of CoS_2_@CP (3.9 mF cm^−2^) and significantly higher than those of MoS_2_@CP (0.41 mF cm^−2^) and CoMoO@CP (0.58 mF cm^−2^).

The long-term catalytic stability of the CoMoS@CP catalyst in 1 M KOH solution was also tested (Fig. 4f). The CoMoS@CP electrode shows the ability to maintain a constant current density of 10 mA cm^−‍2^ for 24-h continuous operation with no increase in the overpotential applied, indicative of the outstanding catalytic stability of the electrocatalyst. It is noteworthy that the overpotential even decreased slightly (ca. 40 mV) during the 24-h test period.

The enhanced OER activity observed in CoMoS@CP compared to its oxide counterpart can be ascribed to the following factors. Transition metal sulphides have high conductivity compared to metal oxides and hydroxides, making them ideal candidates for catalysing OER [70]. While sulphides themselves may not serve as direct active sites, the amorphous metal oxides generated through the initial surface oxidation of metal sulphides during OER offer active sites for O* adsorption [71]. Moreover, previous studies have reported that amorphous metal oxides derived from metal sulphides exhibit improved OER catalytic activity compared to their crystalline counterparts [72]. Given the relative ease of deriving amorphous metal oxides from metal sulphides compared to their crystalline counterparts, metal sulphides are anticipated to be more favourable candidates for OER catalysis [71]. These combined features collectively contribute to the inherently high OER activity of CoMoS@CP.

**Fig. 5:**
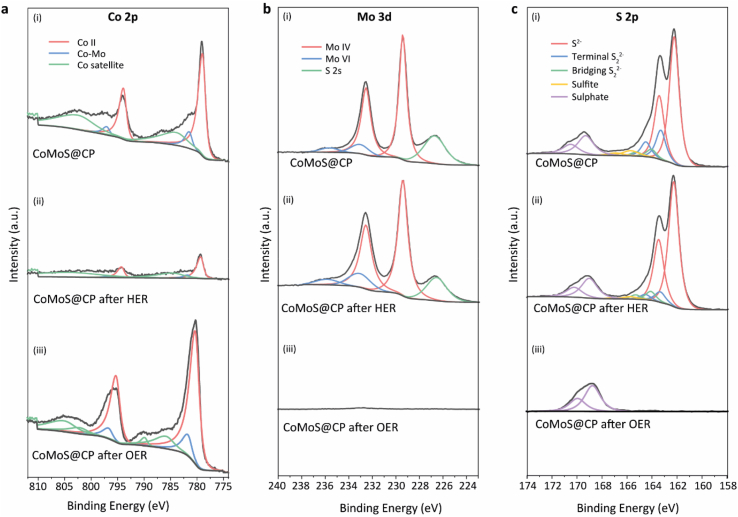
The min-max normalised (a) Co 2*p* (b) Mo 3*d* and (c) S 2*p* spectra of CoMoS after HER test in 0.5 M H_2_SO_4_ and OER test in 1 M KOH.

To gain deeper insights into the HER/OER process, XPS analysis (Fig. 5) and SEM imaging (Fig. S11) was conducted on the same CoMoS@CP electrodes after HER testing in 0.5 M H_2_SO_4_ and OER testing in 1 M KOH.

As shown in Fig. 5a(ii), post-mortem analysis of the Co 2*p* spectrum of CoMoS@CP revealed a reduction in signal intensity compared to Fig. 5a(i), indicating a decrease in Co content after HER. Whereas for the Mo 3*d* spectrum shown in Fig. 5b(i,ii), there is hardly any loss of Mo content after HER. The intensity of the S 2*p* spectrum in Fig. 5c(i,ii) exhibits a slight decrease after HER, primarily due to the reduced peak intensity of the terminal S_2_^2−^ state originating from the Co–Mo–S interfaces. It is worth noting that despite the observed performance drop of about 60 mV in Fig. 4c, the activity remains high after a 24-h catalytic stability test in0.5 M H_2_SO_4_. This implies that, even with the dissolution of Co species, the pivotal catalytic active sites contributing to HER are retained on the catalyst surface.

For the opposite half-cell, a shift of the chemical state of the main peak in the Co *2p* spectrum to a higher valence state (1.3 eV) is observed after experiencing a continuous oxidation current during the OER test (Fig. 5a(i,iii)), which implies that cobalt (oxy)hydroxides are formed from CoS_2_ during the OER process [26]. As shown in Fig. 5b(i,iii), the molybdenum content after the prolonged test decreased from 12 At% to 0.5 At%. In addition, as shown in Fig. 5c(i,iii), the S content also decreased from 36 At% to 4 At%, which is mainly due to the diminishing of sulphides and disulphides states. These observations suggest that the newly formed cobalt (oxy)hydroxides during the OER process mainly contributed to the enhancement of the electrocatalytic activity [26,52,67], and hence the observed loss of Mo species does not influence OER catalysis. Due to this simultaneous OER and oxidation of cobalt species, a gradual enhancement of OER performance was observed as revealed in Fig. 4f [66,67,70].

### 3.3 Overall water electrocatalysis in alkaline electrolyte

**Fig. 6:**
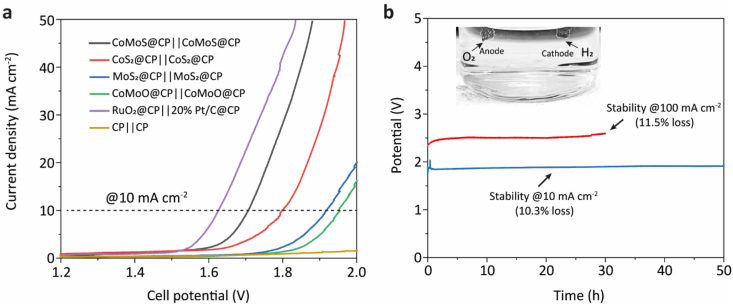
(a) Two electrode OER polarisation curves of CoMoS@CP||CoMoS@CP and control samples in 1 M KOH for overall water splitting ability test. (b) Chronopotentiometry scan at 10 mA cm^−2^ for 50 h and at 100 mA cm^−2^ for 30 h to test the long-term catalytic stability of CoMoS@CP as both anode and cathode towards overall water splitting. Inset: electrode image of O_2_/H_2_ evolution.

Having demonstrated outstanding electrocatalytic ability for both HER and OER, the electrochemical bifunctionality of CoMoS@CP for overall water splitting was examined. The self-supported CoMoS@CP material was used as both anode and cathode (CoMoS@CP ǁ CoMoS@CP) in a two-electrode electrolyser system and performed the electrochemical measurements in the alkaline electrolyte (1 M KOH). In comparison, electrochemical measurements were also performed using electrode pairs of CoS_2_@CP ǁ CoS_2_@CP, MoS_2_@CP ǁ MoS_2_@CP, CoMoO@CP ǁ CoMoO@CP and blank CP ǁ CP as well as coupled electrodes of RuO_2_@CP ǁ 20 % Pt/C@CP as the standard reference. All samples prepared by AACVD were synthesized with an overall mass loading of 0.3 mmol precursors during the deposition process. As shown in Fig. 6a, the CoMoS@CP electrode-pair showed remarkable electrocatalytic activity among the as-synthesized catalysts with a cell voltage of 1.70 V at 10 mA cm^−2^, which implies a combined 470 mV of overpotential for this catalyst. This voltage is remarkably low and is very close to that of the benchmark, RuO_2_@CP ǁ 20 % Pt/C@CP (ca. 1.63 V) and other recently reported noble-metal-free bifunctional electrocatalysts (Table S4). Notably, the loading amount of CoMoS composites for each electrode were merely 0.2 mg cm^−2^ to produce such an excellent activity, while other reported catalysts generally need 5 to 50-fold higher loadings [29]. The cell voltages for CoS_2_@CP, MoS_2_@CP, and CoMoO@CP electrode pairs were 1.80, 1.92, and 1.95 V, significantly higher than CoMoS@CP sample. The bare CP electrodes showed unnoticeable electrochemical activity (2.5 V of cell voltage at 10 mA cm^−2^), which guarantees a minimal contribution of the blank substrate. The catalytic stability was also tested for the CoMoS@CP ǁ CoMoS@CP electrode pair for overall water splitting. As shown in Fig. 6b, at a constant current density of 10 mA cm^−2^, 10.3 % deterioration in the applied voltage and continuous generation of H_2_ and O_2_ bubbles can be observed for a 50-h operation. To further demonstrate the catalytic stability at near practical conditions, the CoMoS@CP electrodes were operated at a large current density of 100 mA cm^−2^ and showed only 11.3 % performance loss after a 30-h test.

The enhanced electrocatalytic performance of the CoMoS@CP is attributed to the main following reasons: (i) the *in-situ* AACVD-grown CoMoS nanocomposites on the CP not only stabilises the catalyst during the electrochemical reactions, but also facilitates the mass/charge transport and electron transfer between the catalyst and electrode; (ii) charge transfer between CoS_2_ to MoS_2_ when forming the heterostructure along with the Co–Mo–S interface of the nanocomposites changes the electronic environment of Co, Mo, and S (as revealed by XPS data in Fig. 3d–f), which favours the adsorption of H^+^ and OH^−^ with the catalyst, resulting in a synergistic enhancement of HER and OER activity [43,56,57,73], (iii) the nanocrystalline/amorphous nature of the CoMoS nanocomposites grown on CP possesses more active defects than those of crystalline structures, thus further increasing the electrochemical activity [12,74,75]. Consequently, these factors in combination are responsible for the improvement of HER and OER activity of the CoMoS@CP electrocatalysts.

## 4 Conclusions

In summary, we have reported a novel AACVD-aided *in-situ* synthesis of CoMoS nanocomposites on carbon paper as electrodes for bifunctional electrochemical water splitting. This electrocatalyst has demonstrated improved HER and OER activities due to the rich active sites and defects, strong interfacial interaction, enhanced conductivity, fast mass transport, and binder-free nature. Further, because the CoMoS was grown *in-situ* on the self-supported CP, it showed exceptional long-term catalytic stability in HER, OER, as well as in overall water electrocatalysis. In addition, the fabrication of the CoMoS@CP electrode assisted by AACVD is relatively simple, controllable, and easily scalable, facilitating potential applications in industrial-scale manufacture. These advantages have therefore made our synthetic CoMoS@CP nanocomposites a promising alternative to the non-precious electrocatalysts for bifunctional water splitting.

## Data availability

Data will be made available on request.

## CRediT authorship contribution statement

**Yuting Yao:** Writing – original draft, Methodology, Investigation, Formal analysis, Data curation, Conceptualization. **Yuhan Liu:** Writing – review & editing, Investigation, Formal analysis. **Juhun Shin:** Writing – review & editing, Conceptualization. **Shenglin Cai:** Writing – review & editing, Visualization. **Xinyue Zhang:** Methodology. **Zhengxiao Guo:** Writing – review & editing, Supervision. **Christopher S. Blackman:** Writing – review & editing, Supervision, Resources, Project administration, Methodology, Conceptualization.

## Declaration of competing interest

The authors declare the following financial interests/personal relationships which may be considered as potential competing interests: Juhun Shin reports a relationship with 10.13039/501100000266Engineering and Physical Sciences Research Council that includes: funding grants. Juhun Shin reports a relationship with 10.13039/100016320Keysight Technologies Inc that includes: funding grants. Yuhan Liu reports a relationship with 10.13039/501100004543China Scholarship Council that includes: funding grants. If there are other authors, they declare that they have no known competing financial interests or personal relationships that could have appeared to influence the work reported in this paper.
