# Supplementary material for: In-situ fabrication of self-supported cobalt molybdenum sulphide on carbon paper for bifunctional water electrocatalysis
Source: Heliyon. 2024 May 10;10(10):e31108. doi: 10.1016/j.heliyon.2024.e31108 (PMC11141360; doi:10.1016/j.heliyon.2024.e31108)
Supplement: Multimedia component 1 [file mmc1.docx]

Supporting Information

*In-situ* fabrication of self-supported cobalt molybdenum sulphide on carbon paper for bifunctional water electrocatalysis

Yuting Yao^a^, Yuhan Liu^a^, Juhun Shin^a^, Shenglin Cai^b^, Xinyue Zhang^a^, Zhengxiao Guo^a,c,d,^*, Christopher S. Blackman^a,^*

*^a^Department of Chemistry, University College London, 20 Gordon Street, London, WC1H 0AJ, UK*

*^b^Yusuf Hamied Department of Chemistry, University of Cambridge, Lensfield Road, Cambridge, CB2 1EW, UK*

*^c^Department of Chemistry, HKU-CAS Joint Laboratory on New Materials, University of Hong Kong, Hong Kong SAR 999077, China*

*^d^HKU Zhejiang Institute of Research and Innovation, Hangzhou 311305, China*

*Corresponding authors.

*Email addresses:* [c.blackman@ucl.ac.uk](mailto:c.blackman@ucl.ac.uk) (C. Blackman), [zxguo@hku.hk](mailto:zxguo@hku.hk) (Z. Guo).

**Chemicals and materials**

Carbon paper, cobalt(II) acetylacetonate (99%, ACROS Organics), molybdenum hexacarbonyl (98%, ACROS Organics), sulphur (99.98%, Sigma-Aldrich), nitrogen (oxygen-free, BOC), sulphuric acid (96%, ACROS Organics), potassium hydroxide pellets (VWR International), methanol (VWR International), acetone (VWR Internation10al), 20% platinum on carbon black (20% Pt/C) (Alfa Aesar), and RuO_2_ (99.9%, Sigma-Aldrich).

**Supplementary Figures**


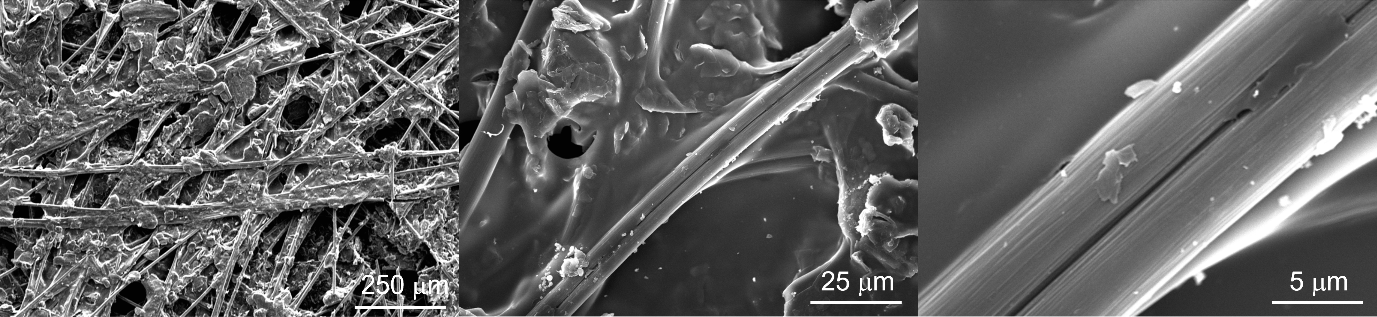


**Figure S1** SEM images of blank carbon paper at various magnifications.


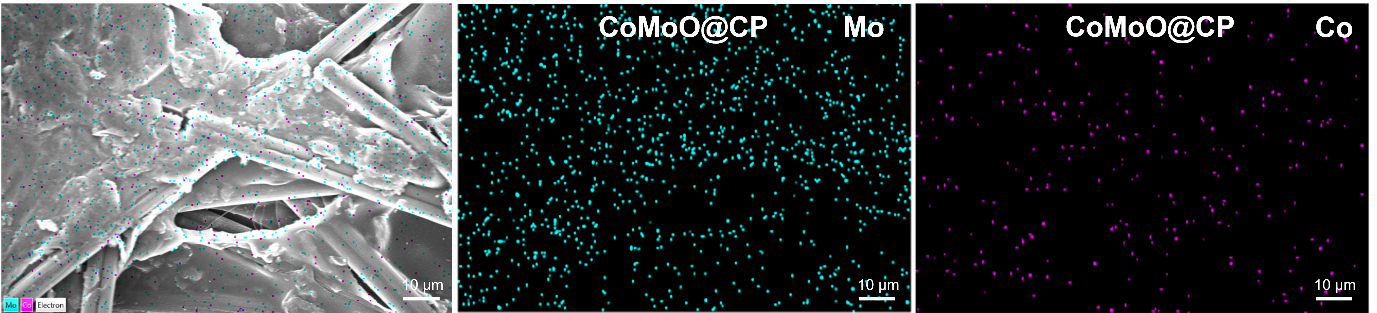


**Figure S2** EDS mapping of intermediate CoMoO@CP.

**
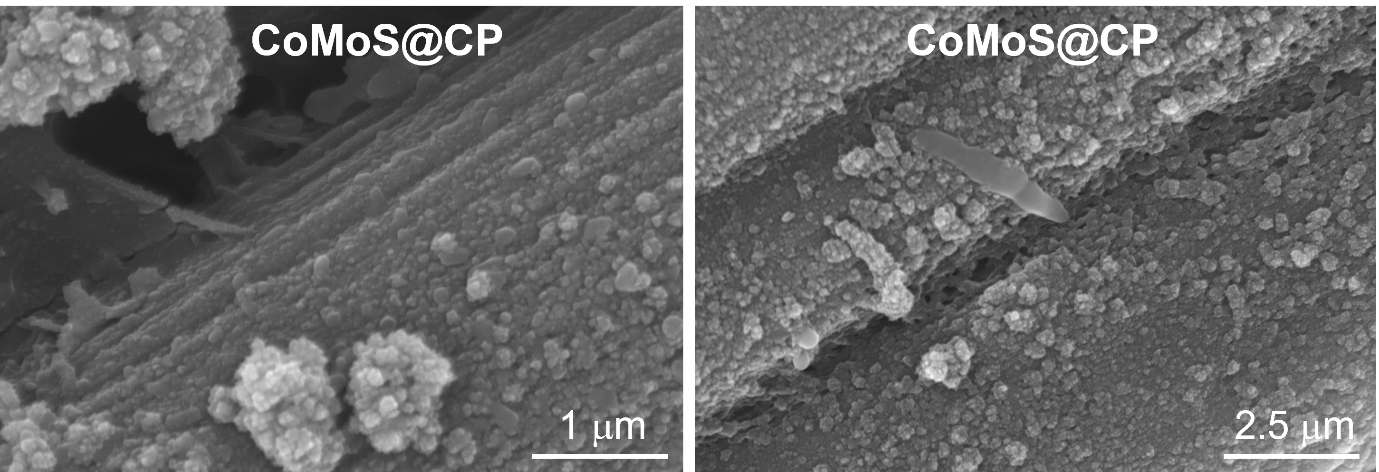
**

**Figure S3** SEM images of CoMoS@CP at various magnifications.


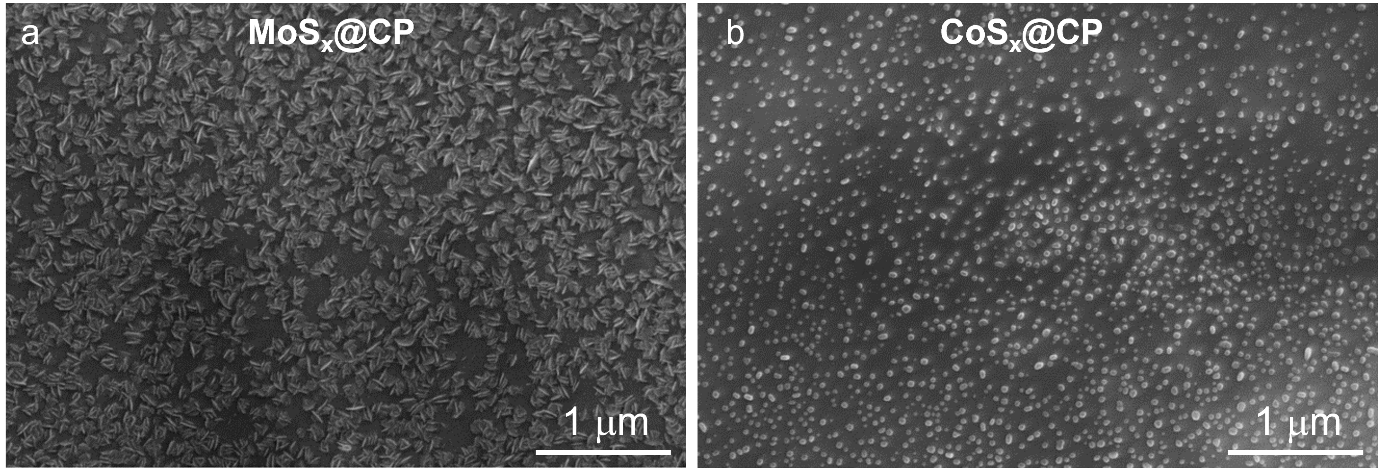


**Figure S4** SEM images of (a) MoS_x_@CP and (b) CoS_x_@CP at lower magnification.


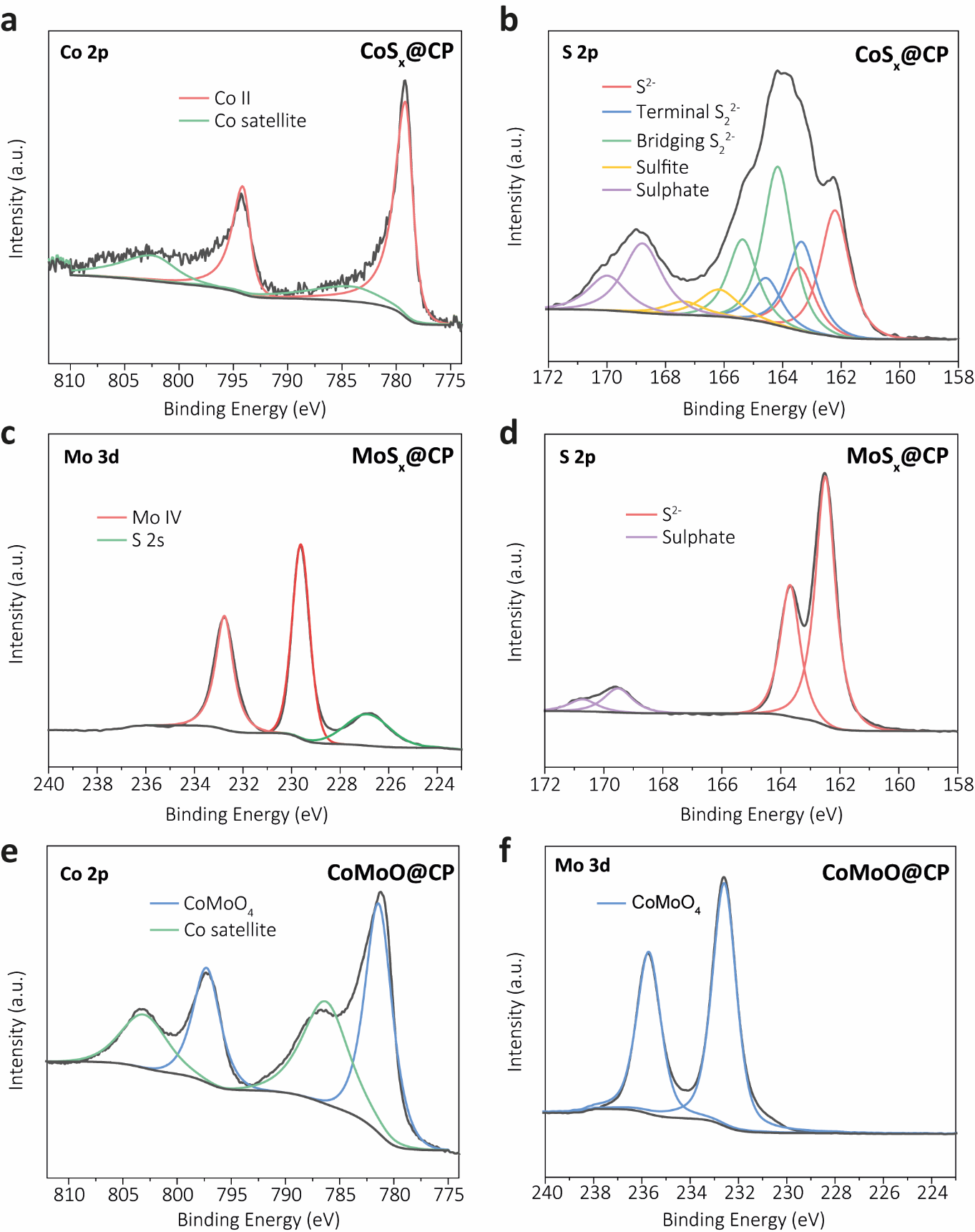


**Figure S5** XPS high-resolution (a) Co 2*p* and (b) S 2*p* spectra for CoS_x_@CP; (c) Mo 3*d* and (d) S 2*p* spectra for MoS_x_@CP; (e) Co 2*p* and (f) Mo 3*d* spectra for CoMoO@CP.


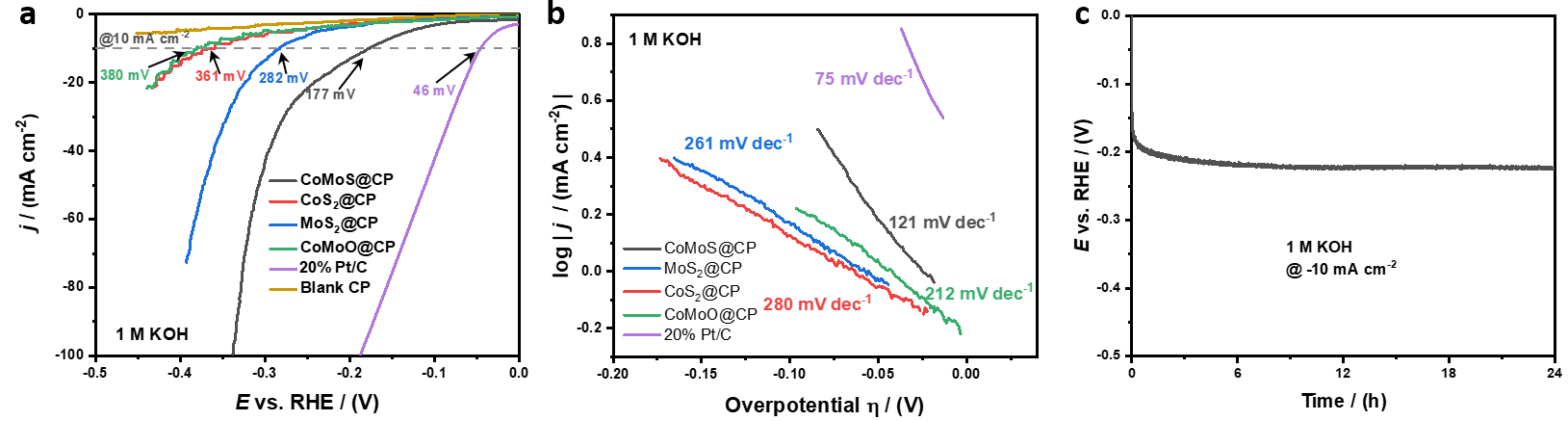


**Figure S6** (a) Electrocatalytic HER polarisation curves of CoMoS@CP and control samples in 1 M KOH and (b) the corresponding Tafel plots. (c) Chronopotentiometry scan at -10 mA cm^-2^ for 24 hours to test the long-term stability of CoMoS@CP for HER in 1 M KOH.

**Electrochemical impedance spectroscopy and electrochemical surface area**

The electrochemical impedance spectroscopy (EIS) measurements were carried out at -0.4 V vs. Ag/AgCl in 0.5 M H_2_SO_4_ and 0.55 V vs. Ag/AgCl in 1 M KOH with changing frequencies from 100 kHz to 0.1 Hz with 10 points per decade and AC voltage of 5 mV rms. The double layer capacitance (C_dl_) was obtained by recording CVs in the non-faradaic region at scan rates between 10 and 100 mV s^-1^ with 10 mV s^-1^ increments. The electrochemical surface area (ECSA) was estimated using equation ECSA =C_dl_/C_s_, where C_s_ is specific capacitance = 0.04 mF per ECSA.


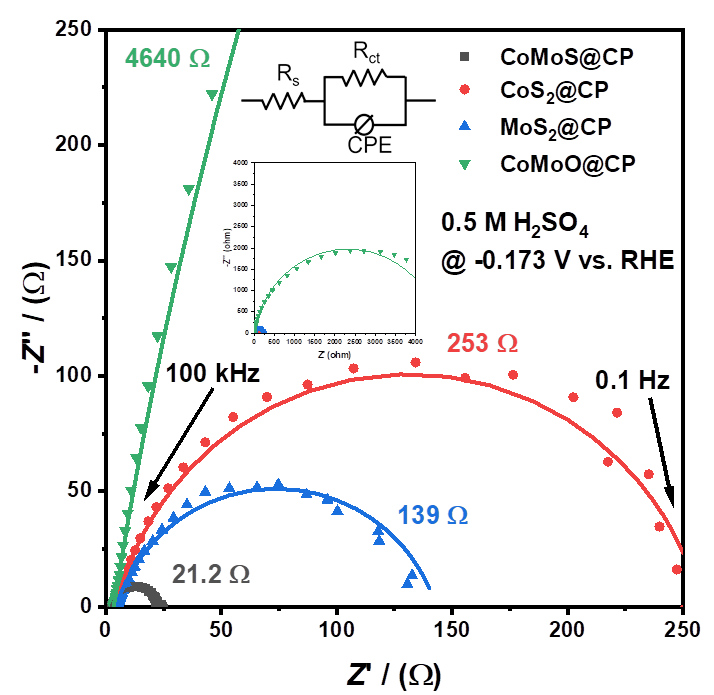


**Figure S7** Nyquist plots and corresponding R_ct_ of CoMoS@CP and control samples at -0.173 V (vs. RHE) in 0.5 M H_2_SO_4_ acquired using electrochemical impedance spectroscopy. Inset: Nyquist plot of CoMoO@CP in full range.


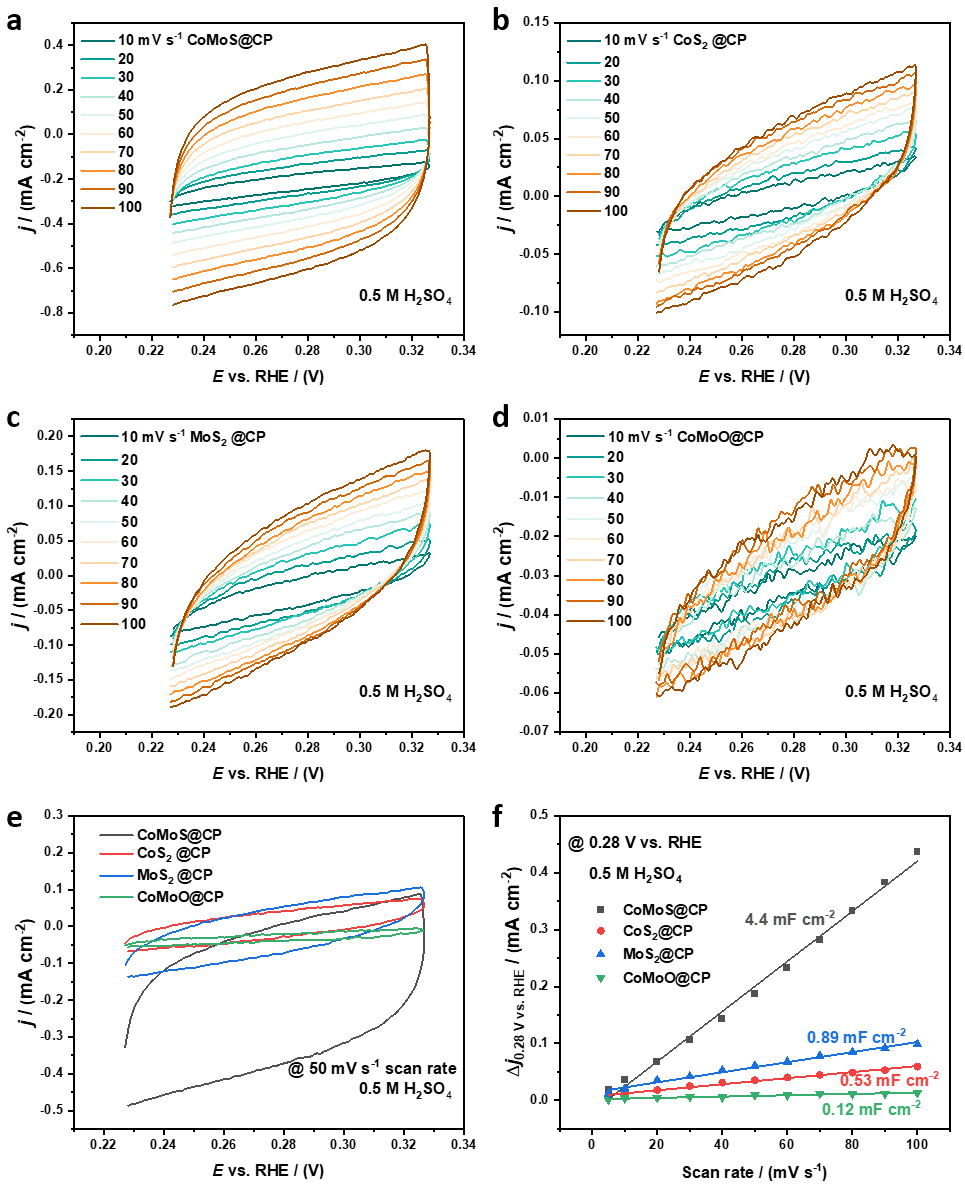


**Figure S8** Cyclic voltammograms of (a) CoMoS@CP, (b) CoS_2_@CP, (c) MoS_2_@CP and (d) CoMoO@CP in the non-faradic region with varying scan rates from 10 to 100 mV s^-1^ in 0.5 M H_2_SO_4_. (e) Cyclic voltammograms of CoMoS@CP and control samples at scan rate of 50 mV s^-1^ in 0.5 M H_2_SO_4_. (f) The average of anodic and cathodic capacitive currents (Δ *j*) at 0.28 V (vs. RHE) with varying scan rates from 10 to 100 mV s^-1^ for CoMoS@CP and control samples in 0.5 M H_2_SO_4_; the corresponding slope of the fitted line is the double layer capacitance (C_dl_) for each sample.


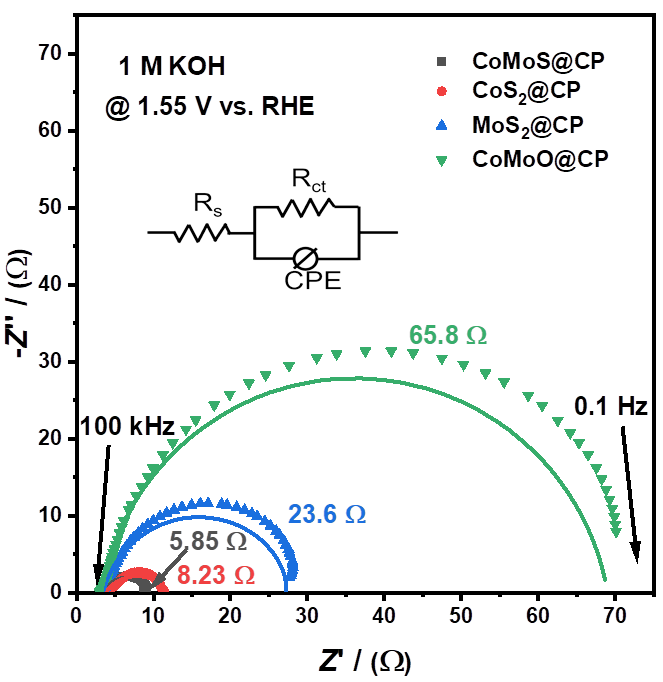


**Figure S9** Nyquist plots and corresponding R_ct_ of CoMoS@CP and control samples at 1.55 V (vs. RHE) in 1 M KOH acquired using electrochemical impedance spectroscopy.


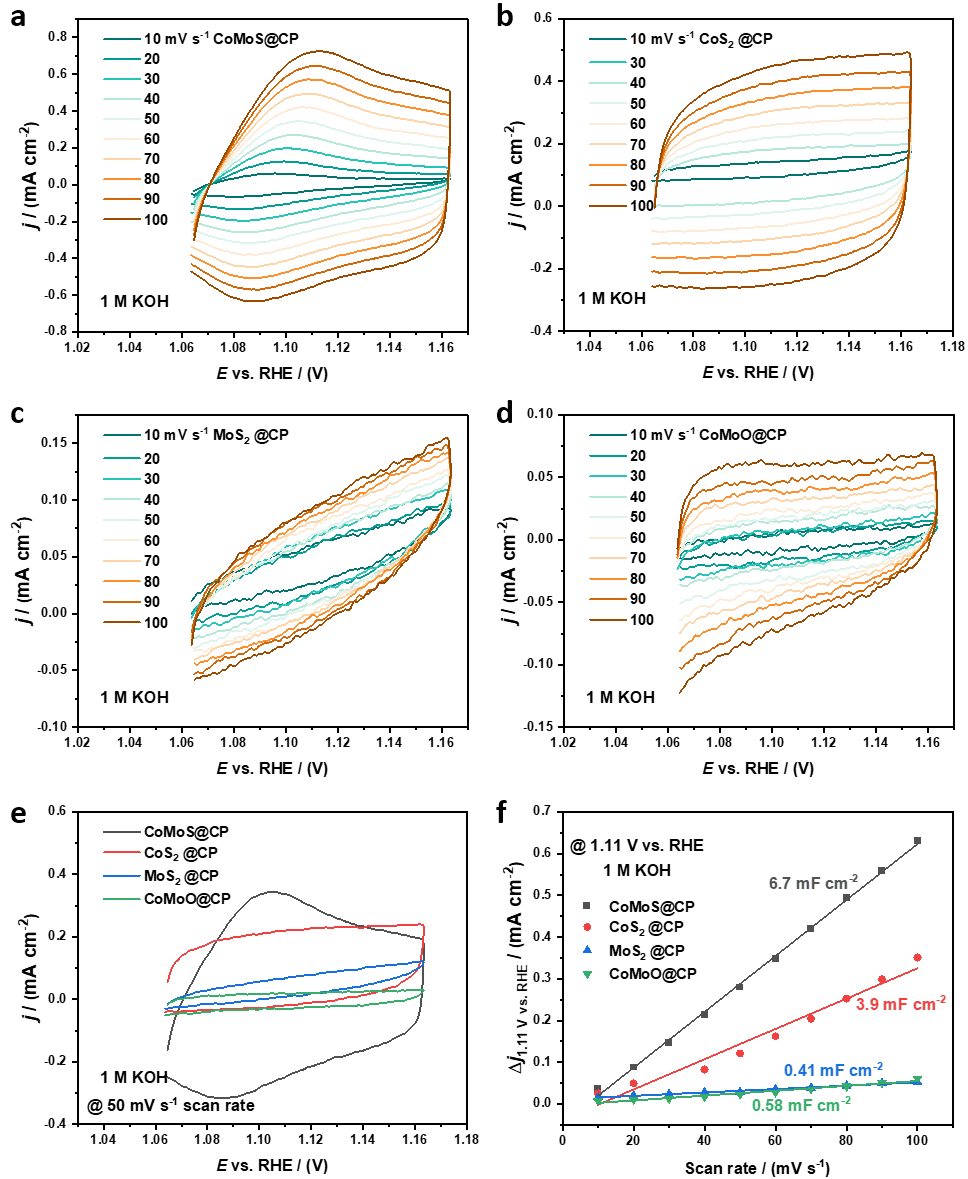


**Figure S10** Cyclic voltammograms of (a) CoMoS@CP, (b) CoS_2_@CP, (c) MoS_2_@CP and (d) CoMoO@CP in the non-faradic region with varying scan rates from 10 to 100 mV s^-1^ in 1 M KOH. (e) Cyclic voltammograms of CoMoS@CP and control samples at scan rate of 50 mV s^-1^ in 1 M KOH. (f) The average of anodic and cathodic capacitive currents (Δ *j*) at 1.11 V (vs. RHE) with varying scan rates from 10 to 100 mV s^-1^ for CoMoS@CP and control samples in 1 M KOH; the corresponding slope of the fitted line is the double layer capacitance (C_dl_) for each sample.


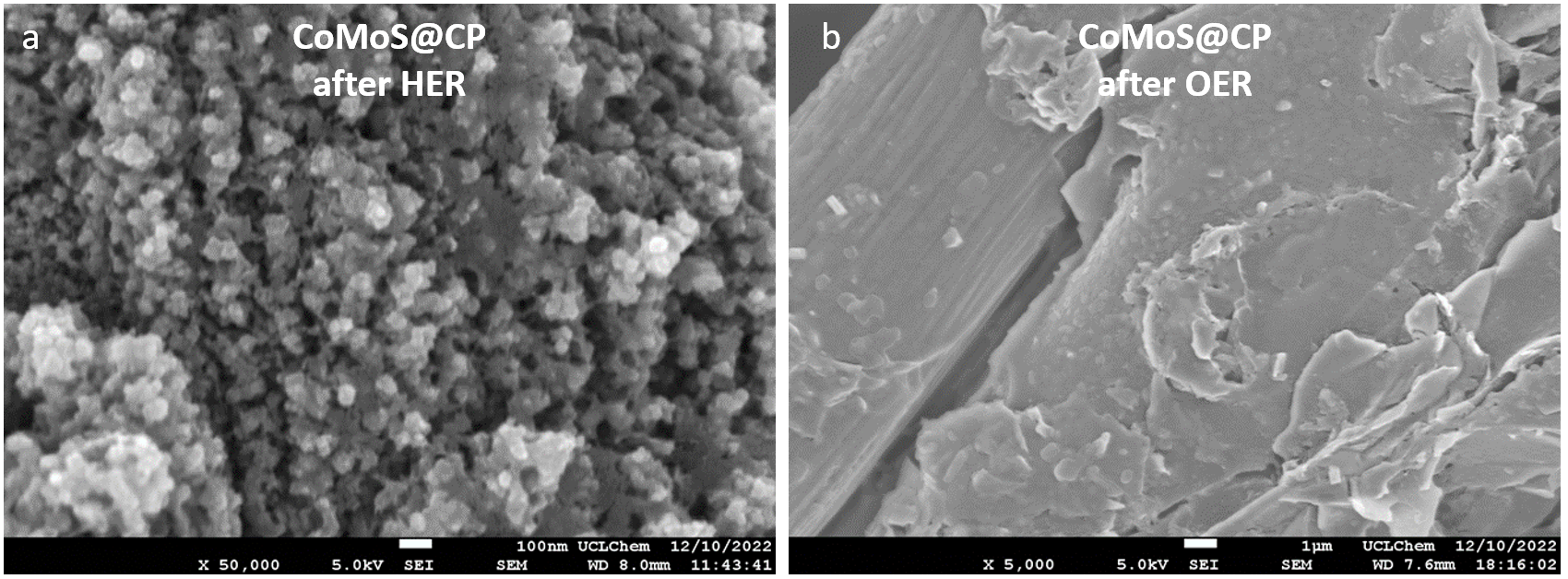


**Figure S11** SEM images of (a) CoMoS@CP after HER and (b) CoMoS@CP after OER.

Table S1 Summary of the electrochemical parameters for the HER in 0.5 M H_2_SO_4_.

| **Materials** | **ŋ_10-HER_/**  **mV** | **ŋ_100-HER_/**  **mV** | **Tafel slope/**  **mV dec^-1^** | **C_dl_/**  **mF cm^-2^** | **ECSA/**  **cm^2^_ECSA_** | **R_ct_/**  **Ω** |
| --- | --- | --- | --- | --- | --- | --- |
| **CoMoS@CP** | 171 | 265 | 75 | 4.4 | 110 | 21.2 |
| **CoS_2_@CP** | 343 | / | 108 | 0.53 | 13 | 253 |
| **MoS_2_@CP** | 265 | / | 93 | 0.89 | 22 | 139 |
| **CoMoO@CP** | / | / | 230 | 0.12 | 3 | 4640 |
| **20% Pt/C** | 42 | 110 | 26 | / | / | / |

Table S2 Comparison of HER activity of CoMoS@CP with similar Co-Mo-S ternary composites reported previously.

| **Materials** | **Mass Loading/**  **mg cm^-2^** | **ŋ_10-HER_/**  **mV** | **ŋ_100-HER_/**  **mV** | **Electrolyte** | **References** |
| --- | --- | --- | --- | --- | --- |
| **CoMoS@CP** | 0.02 | 171 | 265 | 0.5 M H_2_SO_4_ | This work |
| **Co_9_S_8_@MoS_2_/CNFs** | 0.212 | 190 | 250@ŋ_30_ | 0.5 M H_2_SO_4_ | [1] |
| **CoMoS_4_-H** | 0.262 | 170 | N/A | 0.5 M H_2_SO_4_ | [2] |
| **MoS_2_-CoMo_2_S_4_/G** | N/A | N/A | 300@ŋ_85_ | 0.5 M H_2_SO_4_ | [3] |
| **CoMoS@CP** | 0.02 | 177 | 233 | 1 M KOH | This work |
| **CoMoS_4_/NF** | N/A | 141 | N/A | 1 M KOH | [4] |
| **CoMoS_4_/CC** | 1.107 | 143 | 313 | 1 M KOH | [5] |
| **NiCo_2_S_4_ NW/NF** | N/A | 210 | N/A | 1 M KOH | [6] |

Table S3 Summary of the electrochemical parameters for the OER in 1 M KOH.

| **Materials** | **ŋ_10-OER_/**  **mV** | **ŋ_100-OER_/**  **mV** | **Tafel slope/**  **mV dec^-1^** | **C_dl_/**  **mF cm^-2^** | **ECSA/**  **cm^2^_ECSA_** | **R_ct_/**  **Ω** |
| --- | --- | --- | --- | --- | --- | --- |
| **CoMoS@CP** | 409 | 455 | 40 | 6.7 | 168 | 5.85 |
| **CoS_2_@CP** | 420 | 523 | 49 | 3.9 | 98 | 8.23 |
| **MoS_2_@CP** | 445 | 533 | 77 | 0.41 | 10 | 23.6 |
| **CoMoO@CP** | 448 | 530 | 57 | 0.58 | 15 | 65.8 |
| **RuO_2_** | 330 | 485 | 88 | / | / | / |

Table S4 Summary of the cell potentials for the two-electrode overall water splitting in 1 M KOH and comparison with other noble-metal-free bifunctional electrocatalysts.

| **Materials** | **Mass Loading**  **(mg cm^-2^)** | **Cell potential at 10 mA cm^-2^ (V)** | **Stability** | **Reference** |
| --- | --- | --- | --- | --- |
| **CoMoS@CP** | 0.2 | 1.70 | 50 h | This work |
| **Ni_3_S_2_ NA/NF** | 1.6 | 1.76@13 | 150 h | [7] |
| **NiCo_2_S_4_ NA/CC** | 4.0 | 1.68 | 10 h | [8] |
| **NiSe/NF** | 2.8 | 1.63 | 20 h | [9] |
| **MoS_2_/Ni_3_S_2_/NF** | 9.7 | 1.56 | 10 h | [10] |
| **MoS_2_/NiS_2_/CC** | N/A | 1.59 | 24 h | [11] |
| **CoMoS_4_/CC** | 1.107 | 1.72 | 12 h | [5] |
| **NiCo_2_S_4_ NW/NF** | N/A | 1.63 | 50 h | [6] |
| **Ni_0.33_Co_0.6_S NWs/Ti foil** | N/A | 1.65@5 | 20 h | [12] |
| **P-CoMoS/CC** | 2.19 | 1.54 | 100 h | [13] |

**References**

[1] H. Zhu, et al., When Cubic Cobalt Sulfide Meets Layered Molybdenum Disulfide: A Core–Shell System Toward Synergetic Electrocatalytic Water Splitting, Adv. Mater. 27 (32) (2015) 4752-4759.

[2] L. Shao, et al., Low-cost and highly efficient CoMoS_4_/NiMoS_4_-based electrocatalysts for hydrogen evolution reactions over a wide pH range, Electrochim. Acta 213 (2016) 236-243.

[3] X. Zhang, et al., Hybrid catalyst of MoS_2_-CoMo_2_S_4_ on graphene for robust electrochemical hydrogen evolution, Fuel 184 (2016) 559-564.

[4] A.A. Yadav, et al., Ultrasound assisted synthesis of highly active nanoflower-like CoMoS_4_ electrocatalyst for oxygen and hydrogen evolution reactions, Ultrason. Sonochem. 72 (2021) 105454.

[5] Y. Sun, et al., Fabrication of amorphous CoMoS_4_ as a bifunctional electrocatalyst for water splitting under strong alkaline conditions, Nanoscale 8 (45) (2016) 18887-18892.

[6] A. Sivanantham, et al., Hierarchical NiCo_2_S_4_ Nanowire Arrays Supported on Ni Foam: An Efficient and Durable Bifunctional Electrocatalyst for Oxygen and Hydrogen Evolution Reactions, Adv. Funct. Mater. 26 (26) (2016) 4661-4672.

[7] L.-L. Feng, et al., High-Index Faceted Ni_3_S_2_ Nanosheet Arrays as Highly Active and Ultrastable Electrocatalysts for Water Splitting, Journal of the American Chemical Society 137 (44) (2015) 14023-14026.

[8] D. Liu, et al., NiCo_2_S_4_ nanowires array as an efficient bifunctional electrocatalyst for full water splitting with superior activity, Nanoscale 7 (37) (2015) 15122-15126.

[9] C. Tang, et al., NiSe Nanowire Film Supported on Nickel Foam: An Efficient and Stable 3D Bifunctional Electrode for Full Water Splitting, Angew. Chem. Int. Ed. 54 (32) (2015) 9351-9355.

[10] J. Zhang, et al., Interface Engineering of MoS_2_/Ni_3_S_2_ Heterostructures for Highly Enhanced Electrochemical Overall-Water-Splitting Activity, Angew. Chem. Int. Ed. 55 (23) (2016) 6702-6707.

[11] J. Lin, et al., Defect-Rich Heterogeneous MoS_2_/NiS_2_ Nanosheets Electrocatalysts for Efficient Overall Water Splitting, Adv. Sci. 6 (14) (2019) 1900246.

[12] Z. Peng, et al., From Water Oxidation to Reduction: Homologous Ni–Co Based Nanowires as Complementary Water Splitting Electrocatalysts, Adv. Energy Mater. 5 (9) (2015) 1402031.

[13] C. Ray, et al., Amorphous Phosphorus-Incorporated Cobalt Molybdenum Sulfide on Carbon Cloth: An Efficient and Stable Electrocatalyst for Enhanced Overall Water Splitting over Entire pH Values, ACS Appl. Mater. Interfaces 9 (43) (2017) 37739-37749.
